# NGAL/hepcidin-25 ratio and AKI subtypes in patients following cardiac surgery: a prospective observational study

**DOI:** 10.1007/s40620-021-01063-5

**Published:** 2021-05-24

**Authors:** Saban Elitok, Prasad Devarajan, Rinaldo Bellomo, Berend Isermann, Michael Haase, Anja Haase-Fielitz

**Affiliations:** 1 Department of Nephrology and Endocrinology, Ernst Von Bergmann Hospital Potsdam, 14467 Potsdam, Germany; 2 Department of Nephrology and Hypertension, Cincinnati Children’s Hospital, Cincinnati, OH 45229 USA; 3 Department of Intensive Care, Royal Melbourne Hospital, Parkville, Melbourne, VIC 3052 Australia; 4 Department of Intensive Care, Austin Health, Heidelberg, Melbourne, VIC 3084 Australia; 5 Center for Integrated Critical Care, The University of Melbourne, Melbourne, Australia; 6 Institute of Laboratory Medicine, Clinical Chemistry, and Molecular Diagnostic, Leipzig University Hospital, 04103 Leipzig, Germany; 7 Diaverum AB, Renal Care Center Potsdam, 21532 Malmö, Sweden; 8 Medical Faculty, Otto Von-Guericke-University Magdeburg, Leipziger Str. 44, 39120 Magdeburg, Germany; 9 Brandenburg Medical School Theodor Fontane, 16816 Neuruppin, Germany; 10 Faculty of Health Sciences Brandenburg, Potsdam, Germany; 11 Institute of Integrated Health Care Systems Research and Social Medicine, Otto Von-Guericke-University Magdeburg, 39120 Magdeburg, Germany; 12 Department of Cardiology, Brandenburg Heart Center, Immanuel Hospital, 16321 Bernau, Germany

**Keywords:** Cardiopulmonary bypass, Cardiorenal syndrome, NGAL/hepcidin-25 ratio, Subclinical AKI

## Abstract

**Background:**

Acute kidney injury (AKI) subtypes combining kidney functional parameters and injury biomarkers may have prognostic value. We aimed to determine whether neutrophil gelatinase-associated lipocalin (NGAL)/hepcidin-25 ratio (urinary concentrations of NGAL divided by that of hepcidin-25) defined subtypes are of prognostic relevance in cardiac surgery patients.

**Methods:**

We studied 198 higher-risk cardiac surgery patients. We allocated patients to four groups: Kidney Disease Improving Global Outcomes (KDIGO)-AKI-negative and NGAL/hepcidin-25 ratio-negative (no AKI), KDIGO AKI-negative and NGAL/hepcidin-25 ratio-positive (subclinical AKI), KDIGO AKI-positive and NGAL/hepcidin-25 ratio-negative (clinical AKI), KDIGO AKI-positive and NGAL/hepcidin-25 ratio-positive (combined AKI). Outcomes included in-hospital mortality (primary) and long-term mortality (secondary).

**Results:**

We identified 127 (61.6%) patients with no AKI, 13 (6.6%) with subclinical, 40 (20.2%) with clinical and 18 (9.1%) with combined AKI. Subclinical AKI patients had a 23-fold greater in-hospital mortality than no AKI patients. For combined AKI vs. no AKI or clinical AKI, findings were stronger (odds ratios (ORs): 126 and 39, respectively). After adjusting for EuroScore, volume of intraoperative packed red blood cells, and aortic cross-clamp time, subclinical and combined AKI remained associated with greater in-hospital mortality than no AKI and clinical AKI (adjusted ORs: 28.118, 95% CI 1.465–539.703; 3.737, 95% CI 1.746–7.998). Cox proportional hazard models found a significant association of biomarker-informed AKI subtypes with long-term survival compared with no AKI (adjusted ORs: pooled subclinical and clinical AKI: 1.885, 95% CI 1.003–3.542; combined AKI: 1.792, 95% CI 1.367–2.350).

**Conclusions:**

In the presence or absence of KDIGO clinical criteria for AKI, the urinary NGAL/hepcidin-25-ratio appears to detect prognostically relevant AKI subtypes.

**Trial registration number:**

NCT00672334, clinicaltrials.gov, date of registration: 6th May 2008, https://clinicaltrials.gov/ct2/show/NCT00672334.

**Graphic abstract:**

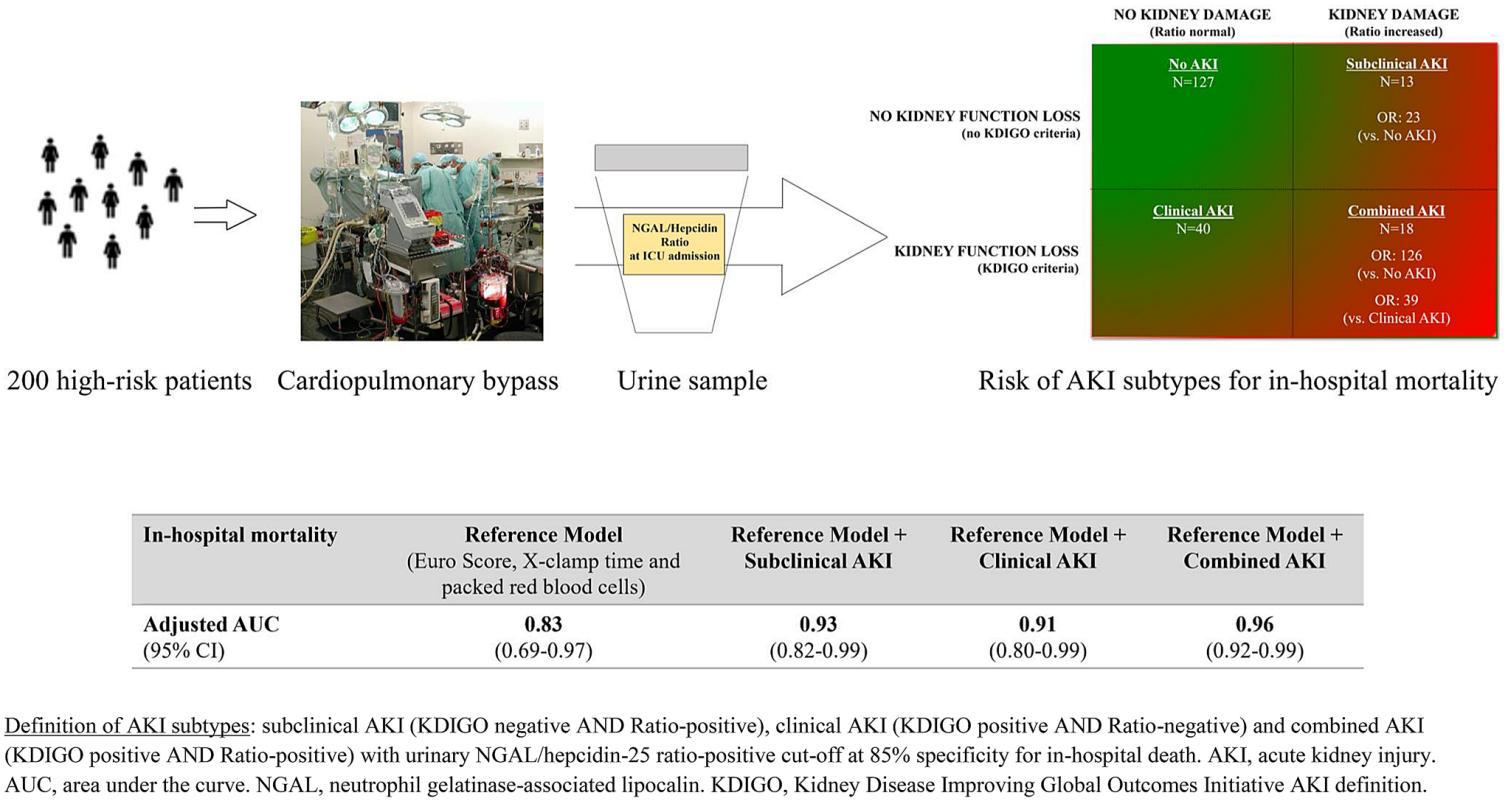

Definition of AKI subtypes: subclinical AKI (KDIGO negative AND Ratio-positive), clinical AKI (KDIGO positive AND Ratio-negative) and combined AKI (KDIGO positive AND Ratio-positive) with urinary NGAL/hepcidin-25 ratio-positive cut-off at 85% specificity for in-hospital death. AKI, acute kidney injury. AUC, area under the curve. NGAL, neutrophil gelatinase-associated lipocalin. KDIGO, Kidney Disease Improving Global Outcomes Initiative AKI definition.

**Supplementary Information:**

The online version contains supplementary material available at 10.1007/s40620-021-01063-5.

## Introduction

Acute kidney injury (AKI) is an independent risk factor for morbidity and mortality after cardiac surgery [1]. Although acute kidney injury events, such as hypoxia, inflammation or toxins like catalytic iron may harm different kidney compartments (proximal/distal tubules, peritubular capillaries), consensus Kidney Disease Improving Global Outcomes (KDIGO) [2] criteria for AKI are still based on filtration-function related parameters (serum creatinine and urine output). Recently, however, serum creatinine-independent kidney biomarkers have been investigated to help improve AKI diagnosis and prognosis. The combination of classical renal function parameters with biomarker levels, e.g., kidney injury molecule-1, interleukin 6, midkine and neutrophil gelatinase-associated lipocalin (NGAL), has led to the introduction of the concept of biomarker-based subtypes of AKI [3–9]. Some of these biomarkers may be particularly relevant to cardiac surgery patients undergoing cardiopulmonary bypass.

The use of cardiopulmonary bypass is associated with inflammation, hemolysis and the release of catalytic iron. Catalytic iron leads to the formation of injurious free radicals [10]. NGAL and hepcidin-25 are released by tubular epithelial cells in response to such catalytic iron production and implicate catalytic iron-mediated mechanisms in human AKI after cardiac surgery [11]. Recently, urinary concentrations of NGAL divided by that of hepcidin-25 (NGAL/hepcidin-25 ratio) were reported to predict major adverse kidney events in patients early after cardiac surgery [12]. Therefore, the NGAL/hepcidin-25 ratio may be particularly useful in defining AKI subtypes in cardiac surgery patients.

Accordingly, we aimed to test the hypothesis of whether an increased NGAL/hepcidin-25-ratio (urinary concentrations of NGAL divided by that of hepcidin-25) at ICU admission can be used to define AKI subtypes in cardiac surgery patients that carry specific and different associations with subsequent in-hospital and long-term mortality.

## Methods

The current study is a prospective observational study using samples from a previously approved, randomized, multicenter study [13], the BICARBONATE study (NCT00672334, clinicaltrials.gov) conducted from May 2008 to June 2011. The previous randomized study involved patients at increased risk of AKI who underwent elective open-heart surgery with the use of cardiopulmonary bypass (Supplemental Fig. 1). The institutional ethics committees granted permission to collect data, conduct biomarker measurement, and track long-term outcomes, including contact with patients and their physicians for the current study (University of Magdeburg, Magdeburg, Germany No. 61/14, 2014; Charité—University Medicine, Berlin, Germany No. ZS EK 11 654/07). The current study was performed in accordance with the ethical standards of the Declaration of Helsinki. Written informed consent to participate and to publish was obtained from all individual participants included in the study. This manuscript adheres to the ‘Strengthening the Reporting of Observational Studies in Epidemiology’ guidelines (Supplemental Table 1) [14].

### Patients

Full study details have been previously described [13]. In brief, we excluded patients undergoing emergency operations (time between hospital admission to operation < 24 h) or off-pump surgery, patients presenting with advanced chronic kidney disease (serum creatinine > 300 µmol/L) or patients on immunosuppressive medication, patients < 18 years of age, and those enrolled in a conflicting research study.

### Outcome variables and data collection

The primary outcome measure was defined as in-hospital mortality. Secondary outcome was long-term mortality. We collected preoperative, peri-operative and postoperative data from medical records and calculated the EuroScore [15].

### Sample collection

Urine samples were obtained at ICU admission as previously described [14]. NGAL concentration (ng/mL) was measured using an Architect analyzer (Abbott Diagnostics, Abbott Park, IL, USA). Human hepcidin 25-isoforms were measured by competitive ELISA (Intrinsic LifeSciences LLC, La Jolla, CA). The lower limit of hepcidin detection was 5.5 ng/mL. The median coefficient of variation was 10% for intra-assay precision and 6% for inter-assay reproducibility. The urinary NGAL/hepcidin-25 ratio was calculated by dividing urinary NGAL concentrations by urinary hepcidin-25 concentrations. Laboratory investigators were blinded to sample sources and clinical outcome.

### Patient allocation according to urinary NGAL/hepcidin-25 ratio- and AKI-status

Patients were grouped according to their *KDIGO criteria-based AKI status* [2] *during the first seven postoperative days* (present/absent) and their *NGAL/hepcidin-25 ratio status at ICU admission* (above/below 85% specificity cut-off value for study endpoints). Acute kidney injury was defined according to the criteria of the KDIGO Initiative [2] using a > 0.3 mg/dL during the first two postoperative days, a > 50% increase in postoperative serum creatinine from baseline or decline in urine output < 0.5 mL/kg/h for at least six hours, both assessed during the first seven postoperative days or initiation of renal replacement therapy (RRT). Thus, four patient groups resulted:

- KDIGO-based AKI-negative and NGAL/hepcidin-25 ratio-negative (no AKI)
- KDIGO-based AKI-negative and NGAL/hepcidin-25 ratio-positive (subclinical AKI)
- KDIGO-based AKI-positive and NGAL/hepcidin-25 ratio-negative (clinical AKI)
- KDIGO-based AKI-positive and NGAL/hepcidin-25 ratio-positive (combined AKI)

### Patient follow-up

Follow up of patients was done in July 2015. Patient’s vital status was obtained at least 5 years after discharge and cross-referenced when possible. We made telephone calls and sought contact by mail at the patients’ homes and through their physicians, and reviewed hospital and physicians’ records. Survival was recorded, if confirmed by the patient or their contact person.

### Statistical analysis

Study size was predetermined by the size of a previous RCT [13]. Maximum effect and clinical actionability of NGAL and hepcidin-25 test results is expected from measurement early after surgery [16, 17]. The cut-off value of the NGAL/hepcidin-25 ratio was determined from the AUC-ROC curve at a high level of specificity (85%) for mortality. For linear variables, the normal distribution assumption was checked using histograms. In the case of normal distribution, the mean (standard deviation) was reported; otherwise, the median (interquartile range) was given. Logarithmic transformations were applied when necessary. Student’s *t*-test, Mann–Whitney *U* test, Kruskal–Wallis test, *χ*^2^ test, or Fisher’s exact test were used where appropriate. The relationship of AKI subtypes with primary or secondary endpoint was calculated after adjustment for reference model (EuroScore, volume of intraoperative packed red blood cells and aortic cross-clamp time) in a logistic regression, and adjusted AUC values and odds ratios (ORs) with 95% confidence intervals were provided. The association of AKI subtypes with long-term patient survival was assessed using Cox proportional-hazard regression analysis adjusting for reference model. Kaplan–Meier curves were plotted. Differences of curves were evaluated using the log-rank test. Information regarding missing data is provided in the table footnotes. Alpha was set at 0.05 and all tests were 2-tailed. SPSS, version 26.0 (IBM Corp., Armonk, NY, USA) was used for statistical analysis.

## Results

### Patient characteristics according to AKI subtypes

Two hundred patients underwent cardiac surgery at the German Heart Center among 350 patients enrolled in a previous multicenter randomized controlled trial (ClinicalTrials.gov NCT00672334). NGAL and hepcidin-25 concentrations were available for 198 patients. Patient flow through the study is shown in Supplemental Fig. 1. Patient characteristics according to AKI subtypes are shown in Table 1. Comparing all AKI subtypes, patients were similar for gender, body mass index and cardiovascular and pulmonary comorbidities. Patients without AKI were younger and had a lower proportion of chronic kidney disease and lower EuroScore. More severe stages of KDIGO criteria-based AKI were observed in patients with combined AKI compared to those with clinical AKI (Table 1). Pre-operative medication, type of cardiac surgery and intraoperative fluid balance and hemodynamic status were similar among AKI subtypes (Table 2). However, volume of intraoperative packed red blood cells, aortic cross-clamp time and concentrations of plasma lactate and urinary NGAL/hepcidin-25 ratio at the end of surgery were higher in patients with AKI. Length of ventilation/intubation and ICU stay were longer for all AKI subtypes compared to no AKI. Thirteen patients died in-hospital. Fifty-eight patients (29.3%) developed AKI according to the KDIGO criteria. Thirty-one patients (15.7%) had a urinary NGAL/hepcidin-25 ratio ≥ 0.5 (AUC-ROC-based 85% specificity cut-off value for hospital mortality).

**Fig. 1:**
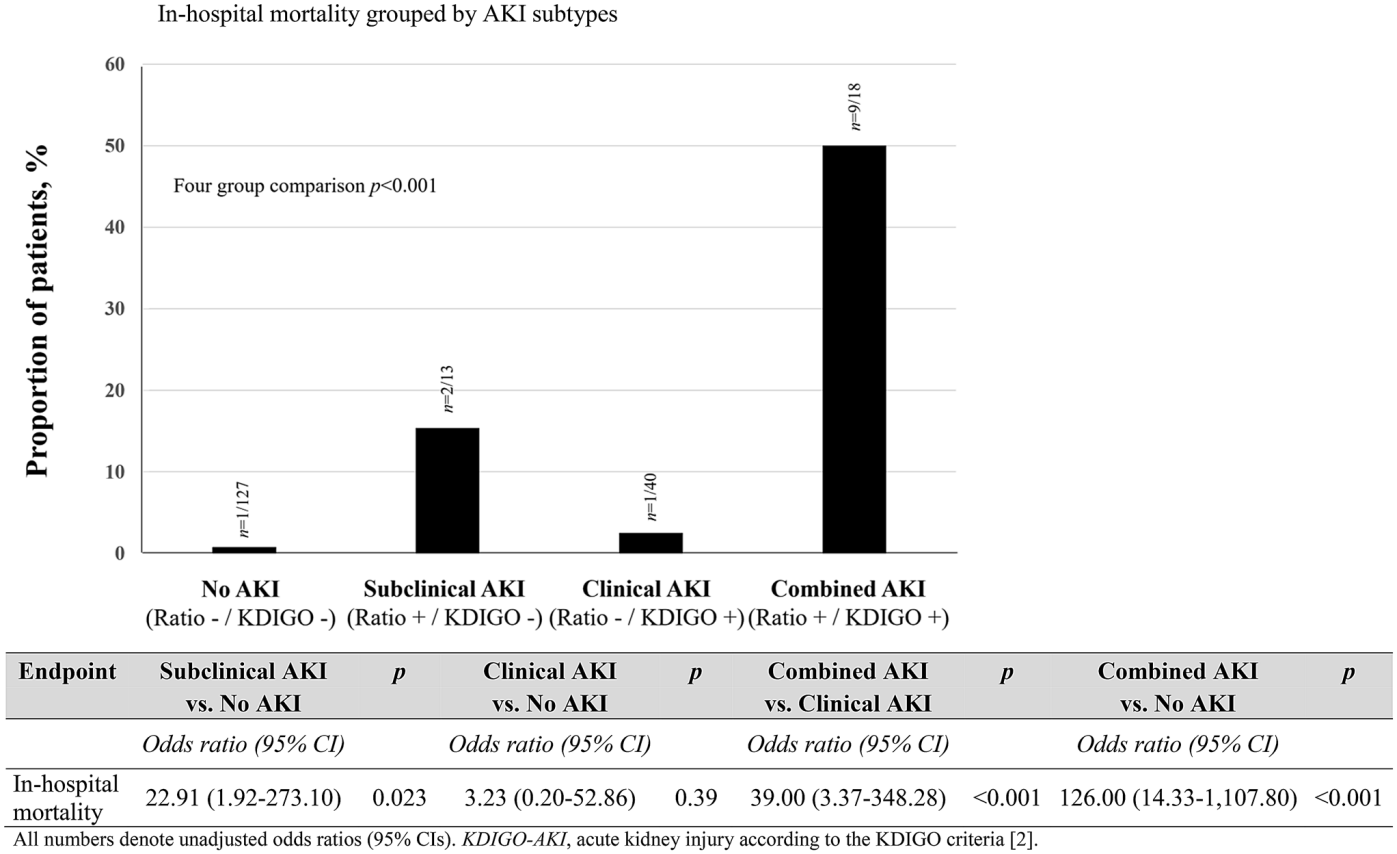
In-hospital mortality grouped by AKI subtypes. Urinary NGAL/hepcidin-25 ratio after end of surgery (–), if ratio < 0.5, NGAL/hepcidin-25 ratio after end of surgery ( +), if ratio ≥ 0.5. Underlying table shows odds ratios and 95% confidence interval (CI) for risk assessment between groups. *NGAL* urine neutrophil gelatinase-associated lipocalin, *KDIGO-AKI* acute kidney injury according to the KDIGO criteria [2]

**Table 1:** Patient characteristics according to status of the urinary NGAL/hepcidin-25 ratio at ICU admission and status of the KDIGO-AKI criteria during the first seven days after surgery

Variable	No AKI *n* = 127 (64.1%)	Subclinical AKI *n* = 13 (6.6%)	Clinical AKI *n* = 40 (20.2%)	Combined AKI *n* = 18 (9.1%)	*p*
Demographic data					
Age (year)	68.0 (56.0–73.0)	74.0 (64.0–80.0)	70.5 (64.0–74.8)	75.0 (68.8–77.3)	0.002
Female	39 (30.7%)	5 (38.5%)	7 (17.5%)	8 (44.4%)	0.154
Body mass index (kg/m^2^)	26.4 (23.5–29.0)	26.5 (23.1–29.2)	27.1 (24.6–30.3)	25.6 (24.5–31.3)	0.537
Comorbidities					
Insulin-dependent diabetes mellitus	6 (5.4%)	0 (0%)	3 (7.5%)	0 (0%)	0.514
Arterial hypertension	94 (74.0%)	10 (76.9%)	33 (82.5%)	16 (89.9%)	0.428
Chronic kidney disease	31 (24.4%)	4 (30.8%)	21 (52.5%)	11 (61.1%)	0.001
Congestive heart failure	21 (16.5%)	3 (23.1%)	13 (32.5%)	3 (16.7%)	0.170
Chronic obstructive pulmonary disease	17 (13.4%)	2 (15.4%)	8 (20.0%)	5 (27.8%)	0.399
Peripheral artery occlusive disease	35 (27.6%)	2 (15.4%)	13 (32.5%)	2 (11.1%)	0.279
EuroScore (points)	6.0 (3.0–7.0)	6.0 (3.5–10.0)	7.0 (5.0–9.8)	8.0 (5.0–10.3)	0.009
Pre-operative medication					
Renin–angiotensin–aldosterone system inhibitors	70 (55.1%)	8 (61.5%)	23 (57.5%)	13 (72.2%)	0.577
Betablockers	91 (71.7%)	11 (84.6%)	30 (75.0%)	13 (72.2%)	0.779
Calcium channel blockers	30 (23.6%)	4 (30.8%)	18 (45.0%)	5 (27.8%)	0.075
Statins	72 (56.7%)	6 (46.2%)	28 (70.0%)	10 (55.6%)	0.357

Values are presented as number (%) or median [interquartile range]. *AKI*, acute kidney injury; *NGAL*, urinary neutrophil gelatinase-associated lipocalin. NGAL/hepcidin-25 ratio positive, if ratio ≥ 0.5

Urinary NGAL/hepcidin-25 ratio at ICU admission and KDIGO-AKI [2] status during the first 7 postoperative days were available for all 198 patients

**Table 2:** Intraoperative characteristics and postoperative outcomes according to status of the urinary NGAL/hepcidin-25 ratio at ICU admission and status of the KDIGO-AKI criteria during the first 7 days after surgery

Variable	No AKI *n* = 127 (64.1%)	Subclinical AKI *n* = 13 (6.6%)	Clinical AKI *n* = 40 (20.2%)	Combined AKI *n* = 18 (9.1%)	*p*
Intraoperative characteristics					
Valve	58 (45.7%)	6 (46.2%)	15 (37.5%)	10 (55.6%)	0.626
Coronary artery bypass graft	23 (18.1%)	1 (7.7%)	9 (22.5%)	2 (11.1%)	0.556
Combined cardiac surgery	35 (27.6%)	5 (38.5%)	10 (25.0%)	4 (22.2%)	0.755
Past cardiac surgery	27 (21.3%)	5 (38.5%)	15 (37.5%)	5 (27.8%)	0.153
Aortic cross-clamp time (min)	72 (55–89)	78 (72–135)	59 (46–96)	120 (65–158)	0.007
Drain output (mL)	150 (50–200)	200 (0–250)	10 (0–200)	0 (0–250)	0.042
Packed red blood cells	0 (0–500)	0 (0–600)	500 (0–750)	750 (400–1000)	< 0.001
Urine output (mL)	1600 (1200–2200)	1500 (900–1800)	1300 (900–1700)	1200 (700–1500)	0.005
Fluid balance (mL)	1300 (400–2200)	800 (200–1600)	1000 (600–2000)	600 (100–2100)	0.333
Furosemide dose, mg	0 (0–0)	0 (0–0)	0 (0–20)	5 (0–25)	0.007
Lowest mean arterial pressure (mmHg)	35 (31–40)	35 (29–42)	33 (28–40)	31 (22–40)	0.145
Vasoconstrictive medication	85 (66.9%)	10 (76.9%)	32 (80.0%)	14 (77.8%)	0.358
Lowest cardiac index (L/min/m^2^)	2.3 (1.8–2.6)	2.1 (2.1–4.5)	2.4 (1.8–2.9)	2.5 (1.8–3.4)	0.448
Laboratory values at ICU admission					
Urinary NGAL/hepcidin-25 ratio	0.009 (0.001–0.030)	0.740 (0.581–1.667)	0.032 (0.004–0.143)	1.010 (0.629–2.690)	< 0.001
Plasma lactate (mmol/L)	1.5 (1.1–3.6)	2.5 (1.9–5.7)	2.0 (1.3–5.0)	3.8 (2.6–7.2)	0.001
Plasma B-type natriuretic peptide (pg/mL)	129.4 (68.1–280.1)	182.3 (43.0–460.0)	242.7 (128.2–381.6)	202.6 (71.6–385.0)	0.301
Plasma C-reactive protein (mg/L)	3.3 (1.9–6.3)	1.5 (0.9–2.9)	3.8 (2.2–9.8)	2.4 (1.9–7.5)	0.547
Postoperative outcomes					
AKI stage^a^					
1	0 (0%)	0 (0%)	33 (82.5%)	5 (27.8%)	< 0.001
2	0 (0%)	0 (0%)	3 (7.5%)	3 (16.7%)	
3	0 (0%)	0 (0%)	4 (10.0%)	10 (55.6%)	
Renal replacement therapy	0 (0%)	0 (0%)	3 (7.5%)	10 (55.6%)	< 0.001
Length of intubation and ventilation (hrs)	18 (12–26)	28 (19–77)	26 (15–520)	74 (34–700)	< 0.001
Length of stay in ICU (h)	25.0 (22.0–46.0)	45.0 (21.0–140.0)	67 (28–270)	155 (46–355)	< 0.001

Values are presented as number (%) or median (interquartile range)

Urinary NGAL/hepcidin-25 ratio at ICU admission and KDIGO-AKI status during the first 7 postoperative days were available for all 198 patients

*AKI*, acute kidney injury; *NGAL*, urinary neutrophil gelatinase-associated lipocalin. NGAL/hepcidin-25 ratio positive, if ratio ≥ 0.5

^a^Highest AKI stage during index hospital admission according to the KDIGO criteria [2]

### AKI subtypes and in-hospital mortality

Overall, 127 (61.6%) patients had no AKI, 13 (6.6%) had subclinical AKI, 40 (20.2%) had clinical AKI and 18 (9.1%) had combined AKI. Patients with subclinical AKI (NGAL/hepcidin-25 ratio ≥ 0.5 but no KDIGO-AKI) had a 23-fold increased risk of in-hospital mortality compared with patients without AKI, almost seven times greater than clinical AKI (Fig. 1). For combined AKI compared to no AKI (126-fold increase in risk) and combined AKI compared to clinical AKI (39-fold increase in risk), the impact was even more pronounced (Fig. 1). Moreover, subclinical and combined AKI were associated with in-hospital mortality compared with no AKI and clinical AKI, respectively (adjusted OR 28.118, 95% CI 1.465–539.703, *p* = 0.027; adjusted OR 3.737, 95% CI 1.746–7.998, *p* = 0.001), even after adjusting for EuroScore, volume of intraoperative packed red blood cells, and aortic cross-clamp time (Supplemental Table 2). The adjusted AUC of the reference model increased from 0.811 to 0.929 or 0.961 after including subclinical or combined AKI, respectively (Supplemental Table 2).

### AKI subtypes and long-term mortality

**Fig. 2:**
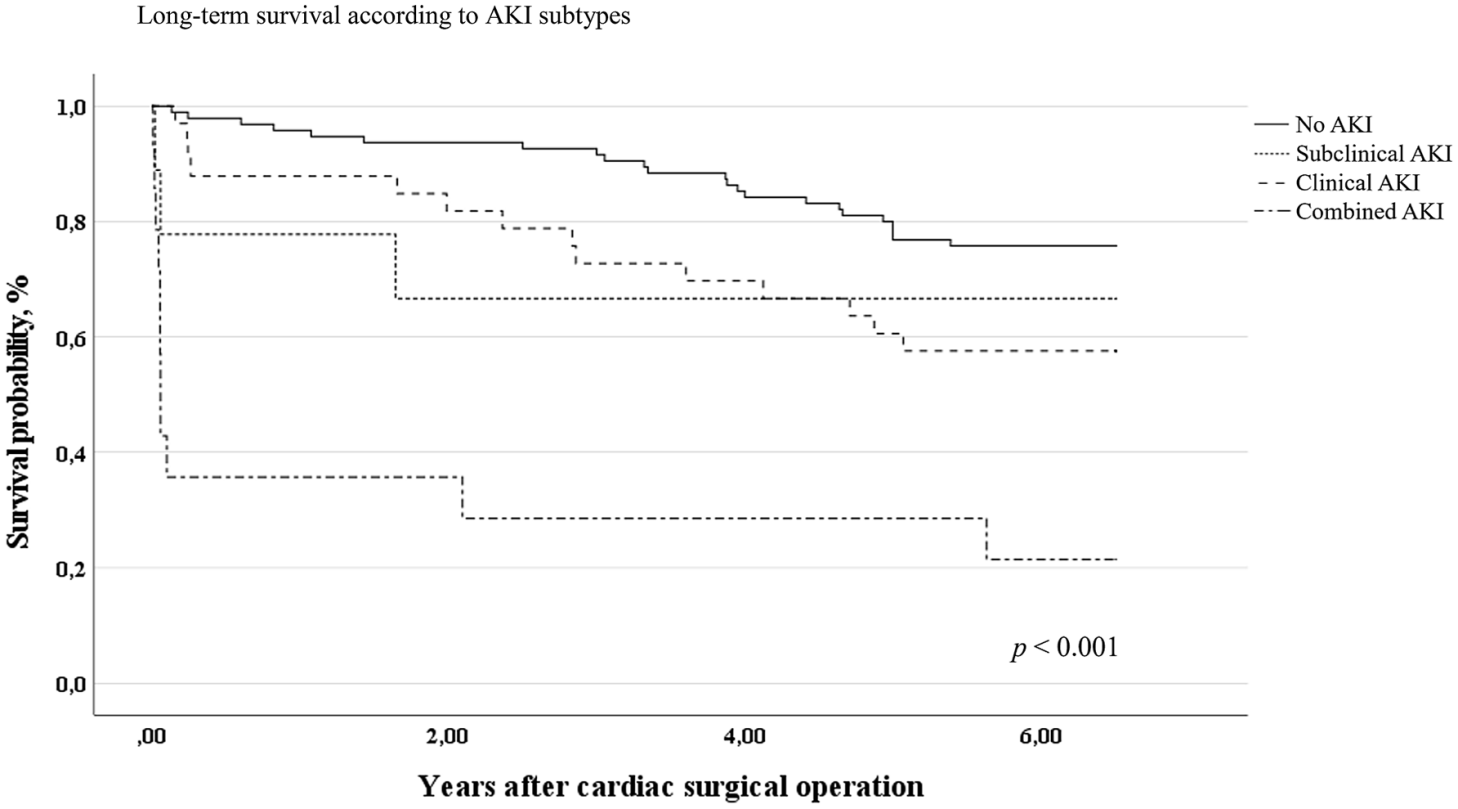
Long-term survival according to AKI subtypes. NGAL/hepcidin-25 ratio and allocated AKI subtype: log-rank test *p* < 0.001. Follow up time was 5.6 years. *NGAL* neutrophil gelatinase-associated lipocalin. Urinary NGAL and hepcidin-25 concentrations and NGAL/hepcidin-25 ratio immediately after end of surgery were available for all 198 patients. *KDIGO-AKI* acute kidney injury according to the KDIGO criteria [2]

Long-term follow-up showed separation of survival according to AKI subtypes (Fig. 2). Subclinical and combined AKI showed higher long-term mortality over 5.6 years of follow-up (14/23 patients [60.9%]) than no AKI and clinical AKI (37/128 patients [28.9%]) (odds ratio 3.826 [95% CI 1.524–9.605], *p* = 0.003).

After adjustment for EuroScore, volume of intraoperative packed red blood cells and aortic cross-clamp time, Cox proportional hazard regression analyses found a significant association of combined AKI (adjusted OR 1.792 [95% CI 1.367–2.350], *p* < 0.001) and pooled subclinical and clinical AKI (adjusted OR 1.885 [95% CI 1.003–3.542], *p* = 0.049) with long-term survival compared with no AKI as reference.

## Discussion

### Key findings

We assessed the urinary NGAL/hepcidin-25 ratio measured at ICU admission alone or combined with clinical functional AKI criteria until postoperative day seven in order to define AKI subtypes following cardiac surgery and to study their relationship with mortality. Subclinical AKI, (high NGAL/hepcidin-25 ratio but no creatinine or urine output criteria for AKI) had a much higher risk of in-hospital mortality than either no AKI or clinical AKI. Its long-term mortality was also higher than in patients with no AKI. The highest mortality rates were observed in patients with combined AKI (9.1% of patients) compared with no AKI or with other forms (subclinical or clinical) of AKI. The study findings remained essentially unchanged after adjustment for clinical risk score or important intraoperative covariates.

A recent systematic review described a state of increased kidney biomarker concentration (NGAL) in the absence of KDIGO creatinine-based criteria for AKI as *‘subclinical AKI’.* Such subclinical AKI was associated with increased length of hospital stay and mortality [18]. However, this was based on a retrospective pooled analysis without details of patient characteristics or adjustment for key covariates. In contrast, Moledina et al. coined the term *‘hemodynamic AKI’* (here *‘clinical AKI’*) to describe a serum creatinine increase in the absence of increased kidney biomarker concentration, as may occur in cardiorenal syndrome or with renin–angiotensin–aldosterone system inhibition [19]. Recently, several original studies have used urinary biomarkers to define AKI subtypes in different patient populations including the Emergency Department, critical care and cardiac settings [3–9]. For example, Albert et al. reported on NGAL and AKI consensus criteria-based AKI subtypes including subclinical (21.1%), clinical (4.5%) and combined AKI (7.5%) in patients following cardiac surgery [5]. However, this study did not include hepcidin-25 nor did it adjust the prognostic value of AKI subtypes for key covariates. None of the previous studies assessed the urinary NGAL/hepcidin-25 ratio for the early detection of AKI subtypes.

The outcome pattern for AKI subtypes observed in our study was similar to that of AKI subtype definition using other urinary biomarkers [5, 9]. Subclinical and clinical AKI showed intermediate outcomes, and combined AKI had worse outcomes compared to patients without AKI. Compared with previous studies, we found a lower incidence of subclinical AKI than that with NGAL (6.6% vs. 21.1% [5]). However, the discriminatory ability of NGAL/hepcidin-25 ratio-defined subclinical AKI was higher [5]. This high discriminatory value may be explained by the role of hepcidin-25 in the metabolism of catalytic iron generated during cardiopulmonary bypass [20, 21]. Hepcidin down-regulates ferroportin and may help decrease free-iron availability in the kidney thereby limiting its toxicity [22]. The observed distinctive outcome pattern supports the notion that the urinary NGAL/hepcidin-25 ratio after surgery may provide prognostic information beyond serum creatinine and urine output. Pre-existing chronic kidney disease was highly prevalent in clinical and combined AKI groups pointing towards the increased risk of creatinine- and urine output-based AKI in these patients, as has been previously described [23]. If compared to subclinical AKI and clinical AKI, we found a higher prevalence of renal replacement therapy/RRT-requiring AKI in the combined AKI group. Whether this finding indicates the potential ability of the urinary NGAL/hepcidin-25 ratio to predict renal replacement therapy/RRT-requiring AKI needs evaluation in further studies. More patients with KDIGO criteria-based, severe AKI were observed in patients with combined AKI compared to those with clinical AKI. Larger prospective studies may address the impact of KDIGO criteria-based severity of AKI in relation to that of biomarker-defined AKI subtypes on mortality.

### Implications of study findings

Our findings imply that the urinary NGAL/hepcidin-25 ratio at the end of surgery, alone or combined with creatinine- or urine output-based criteria, identifies a subpopulation of patients at high mortality risk. Such identification may provide an opportunity for differential therapeutic options [24, 25], and suggests the need to investigate the role of modified follow-up care in such patients [26] and perhaps earlier RRT initiation in those patients with combined AKI [27]. Finally, our findings imply that screening for patients with specific AKI subtypes may reduce the required sample size for the evaluation of interventions in AKI trials [28].

### Strengths and limitations

Our study has several strengths. We investigated typical patients at risk of adverse kidney outcome in a relatively homogeneous and well-defined patient cohort after cardiac surgery. These are the patients for whom allocation to distinct AKI subtypes may be crucial. Study results remained stable after adjustment for established risk scores and other important intraoperative covariates. We acknowledge some limitations. The number of events was limited thereby affecting generalizability of the study results. Hepcidin-25 measurement on a clinical laboratory platform is not yet available, thus large scale application of our research findings to practice is not yet possible. We were not able to provide direct measurement of glomerular filtration rate or histopathological findings of AKI subtypes to better delineate their pathophysiological meaning.

### Conclusion

In conclusion, the urinary NGAL/hepcidin-25-ratio appears to detect prognostically relevant AKI subtypes including subclinical and combined AKI. Cautiously considering the limited number of events in this study, subclinical AKI (no creatinine increase, no urine output decline) appears to detect patients with a type of otherwise undetected clinically highly relevant form of AKI. If confirmed in larger prospective studies, the urinary NGAL/hepcidin-25 ratio might enable the early identification of high-risk patients for future interventional studies.

## Supplementary Information

Below is the link to the electronic supplementary material.

Supplementary file1 (DOCX 97 KB)

## Data Availability

The datasets generated and/or analyzed during the current study are available from the corresponding author on reasonable request.
