# Supplementary material for: NGAL/hepcidin-25 ratio and AKI subtypes in patients following cardiac surgery: a prospective observational study
Source: J Nephrol. 2021 May 24;35(2):597–605. doi: 10.1007/s40620-021-01063-5 (PMC8926978; doi:10.1007/s40620-021-01063-5)

**Supplementary Information for submission to the Journal of Nephrology**

**NGAL/hepcidin-25 ratio and AKI subtypes in patients following cardiac surgery:**

A prospective observational study

Saban Elitok, Prasad Devarajan, Rinaldo Bellomo, Berend Isermann, Michael Haase, Anja Haase-Fielitz

**Corresponding author**

Michael Haase, MD

Otto-von-Guericke University Magdeburg, Medical Faculty

Leipziger Str. 44

39120 Magdeburg

Germany

Tel +49-331-2806911

[michael.haase@med.ovgu.de](mailto:michael.haase@med.ovgu.de)

**Supplemental Table 1.** STROBE Statement - Checklist of items in the present cohort study

|  | Item No | Recommendation | Page |
| --- | --- | --- | --- |
| **Title and abstract** | 1 | (*a*) Indicate the study’s design with a commonly used term in the title or the abstract | 1 |
|  |  | (*b*) Provide in the abstract an informative and balanced summary of what was done and what was found | 3 |
| Introduction | | |  |
| Background/rationale | 2 | Explain the scientific background and rationale for the investigation being reported | 7 |
| Objectives | 3 | State specific objectives, including any prespecified hypotheses | 7 |
| Methods | | |  |
| Study design | 4 | Present key elements of study design early in the paper | 8 |
| Setting | 5 | Describe the setting, locations, and relevant dates, including periods of recruitment, exposure, follow-up, and data collection | 8 |
| Participants | 6 | (*a*) Give the eligibility criteria, and the sources and methods of selection of participants. Describe methods of follow-up | 8 |
|  |  | (*b*) For matched studies, give matching criteria and number of exposed and unexposed | *N/A* |
| Variables | 7 | Clearly define all outcomes, exposures, predictors, potential confounders, and effect modifiers. Give diagnostic criteria, if applicable | 9 |
| Data sources/ measurement | 8* | For each variable of interest, give sources of data and details of methods of assessment (measurement). Describe comparability of assessment methods if there is more than one group | 9 |
| Bias | 9 | Describe any efforts to address potential sources of bias | 10, 11 |
| Study size | 10 | Explain how the study size was arrived at | 10 |
| Quantitative variables | 11 | Explain how quantitative variables were handled in the analyses. If applicable, describe which groupings were chosen and why | 10, 11 |
| Statistical methods | 12 | (*a*) Describe all statistical methods, including those used to control for confounding | 10 |
|  |  | (*b*) Describe any methods used to examine subgroups and interactions | 10 |
|  |  | (*c*) Explain how missing data were addressed | 11 |
|  |  | (*d*) If applicable, explain how loss to follow-up was addressed | Legends to Suppl. Fig. 1 |
|  |  | (*e*) Describe any sensitivity analyses | 11 |
| Results | | |  |
| Participants | 13 | (a) Report numbers of individuals at each stage of study—e.g. numbers potentially eligible, examined for eligibility, confirmed eligible, included in the study, completing follow-up, and analyzed | 12 |
|  |  | (b) Give reasons for non-participation at each stage | 12 |
|  |  | (c) Consider use of a flow diagram | Suppl. Fig. 1 |
| Descriptive data | 14 | (a) Give characteristics of study participants (eg demographic, clinical, social) and information on exposures and potential confounders | 12 |
|  |  | (b) Indicate number of participants with missing data for each variable of interest | Tables 1,2 Fig. legends |
|  |  | (c) Summarize follow-up time (e.g., average and total amount) | 13 |
| Outcome data | 15 | Report numbers of outcome events or summary measures over time | 12 |
| Main results | 16 | (*a*) Give unadjusted estimates and, if applicable, confounder-adjusted estimates and their precision (e.g., 95% confidence interval). Make clear which confounders were adjusted for and why they were included | 12 |
|  |  | (*b*) Report category boundaries when continuous variables were categorized | 12, 13 |
|  |  | (*c*) If relevant, consider translating estimates of relative risk into absolute risk for a meaningful time period | N/A |
| Other analyses | 17 | Report other analyses done—e.g. analyses of subgroups and interactions, and sensitivity analyses | 13 |
| Discussion | | |  |
| Key results | 18 | Summarize key results with reference to study objectives | 14 |
| Limitations | 19 | Discuss limitations of the study, taking into account sources of potential bias or  imprecision. Discuss both direction and magnitude of any potential bias | 16, 17 |
| Interpretation | 20 | Give a cautious overall interpretation of results considering objectives, limitations, multiplicity of analyses, results from similar studies, and other relevant evidence | 15-16 |
| Generalizability | 21 | Discuss the generalizability (external validity) of the study results | 16 |
| Other information | | |  |
| Funding | 22 | Give the source of funding and the role of the funders for the present study and, if applicable, for the original study on which the present article is based | 5 |

**Supplemental Table 2** AUCs and ORs of regression analysis for prediction of in-hospital mortality

|  | **Adjusted AUC**  (95% CI) | ***p*** | **Adjusted odds ratio**  (95% CI) | ***p*** |
| --- | --- | --- | --- | --- |
| **Reference Model** incl.  Euro Score (per point)  Aortic cross-clamp time (per min)  Intraoperative volume of packed red blood cells (per mL) | 0.811 (0.665-0.957) | <0.001 | 1.038 (0.809-1.332)  1.000 (1.000-1.001)  1.001 (0.999-1.003) | 0.767  0.002  0.193 |
| **Reference Model and Subclinical AKI^*^**  Euro Score (per point)  Aortic cross-clamp time (per min)  Intraoperative volume of packed red blood cells (per mL)  Subclinical AKI | 0.929 (0.823-0.999) | 0.011 | 1.068 (0.797-1.432)  1.000 (1.000-1.000)  1.001 (1.000-1.003)  28.118 (1.465-539.703) | 0.660  0.170  0.166  0.027 |
| **Reference Model and Clinical AKI^**^**  Euro Score (per point)  Aortic cross-clamp time (per min)  Intraoperative volume of packed red blood cells (per mL)  Clinical AKI | 0.912 (0.801-0.999) | 0.045 | 0.992 (0.861-1.142)  0.999 (0.999-1.000)  1.002 (0.999-1.005)  1.293 (0.279-6.001) | 0.908  0.101  0.267  0.742 |
| **Reference Model and Combined AKI^***^**  Euro Score (per point)  Aortic cross-clamp time (per min)  Intraoperative volume of packed red blood cells (per mL)  Combined AKI | 0.961 (0.917-0.999) | <0.001 | 1.068 (0.797-1.432)  1.000 (1.000-1.000)  1.001 (1.000-1.003)  3.737 (1.746-7.998) | 0.660  0.170  0.166  0.001 |

^*^Defined as KDIGO criteria [2] negative AND increased urinary NGAL/hepcidin-25 ratio (above cut-off value).

^**^Defined as KDIGO criteria [2] positive AND no increased urinary NGAL/hepcidin-25 ratio (below cut-off value).

^***^Defined as KDIGO criteria [2] positive AND increased urinary NGAL/hepcidin-25 ratio (above cut-off value).

**Supplemental Fig. 1**


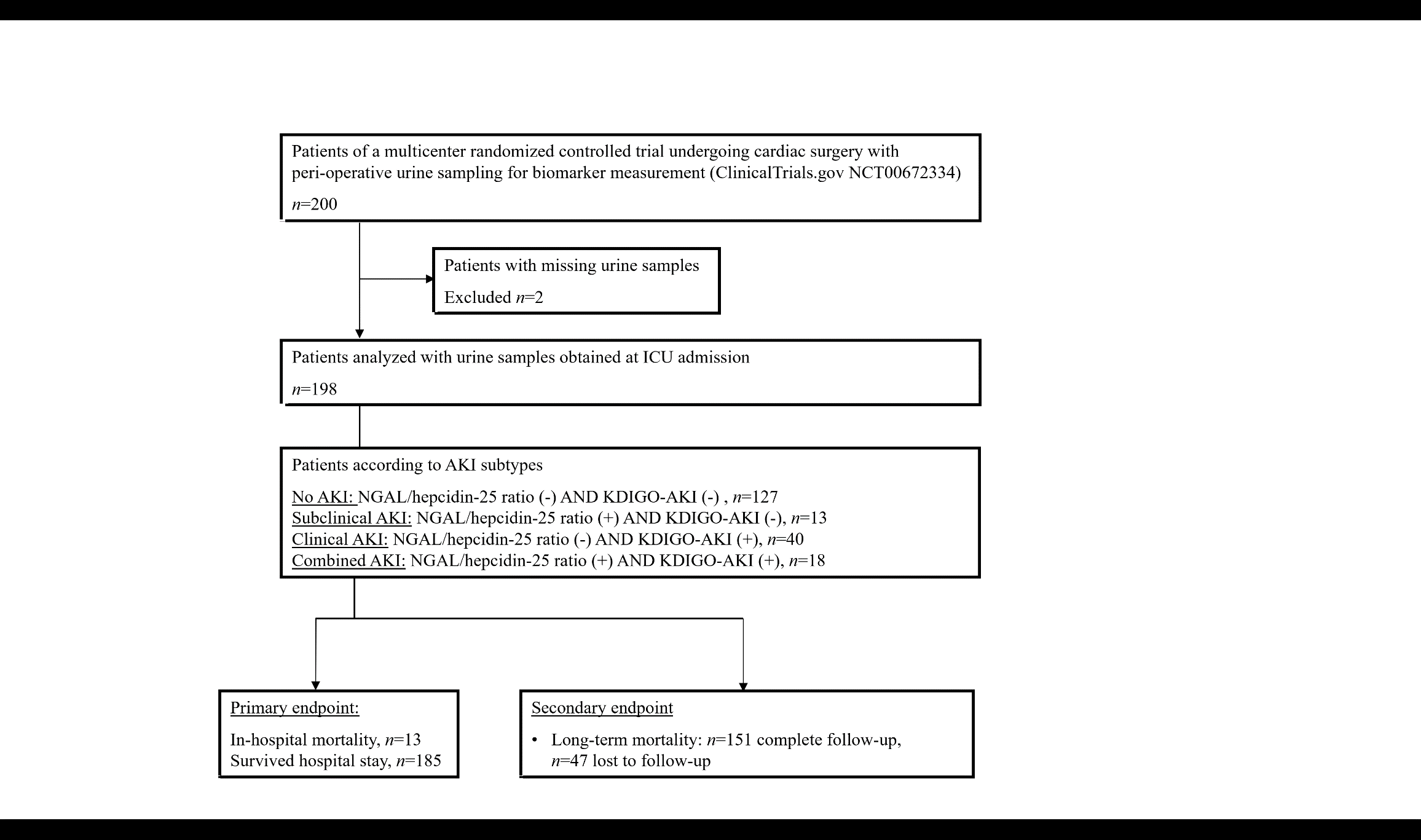

Supplement: Supplementary file 1 — Supplementary file1 (DOCX 97 KB) [file 40620_2021_1063_MOESM1_ESM.docx]
